# 

**Published:** 1911-12-09

**Authors:** 


The following appointments have been made at the General Hospital, Birmingham: Dr. K. D. Wilkinson resident medical officer for one year, and Mr. H. B. Wyman has been re-appointed resident pathologist for six months.

At the Jaffray Branch Hospital, Mr. Oscar Holden, M.B., Ch.B., M.R.C.S., L.R.C.P., has been appointed resident medical and surgical officer for twelve months.

